# Smartphone Addiction and Eysenck's Personality Traits Among Chinese Adolescents: A Meta-Analysis

**DOI:** 10.3389/fpsyg.2021.794112

**Published:** 2022-02-04

**Authors:** Sicheng Xiong, Yi Xu, Bin Zhang, Lihui Zhu, Jianhui Xie

**Affiliations:** ^1^Department of Applied Psychology, Hunan University of Chinese Medicine, Changsha, China; ^2^Department of Rehabilitation, Hunan Children's Hospital, Changsha, China

**Keywords:** adolescent, smartphone addiction, personality traits, meta-analysis, moderators

## Abstract

With the quickly rising popularity of smartphone among adolescents over the past decade, studies have begun to investigate the relationship between smartphone addiction and Eysenck's personality traits. Despite numerous studies on this topic, however, findings have been mixed and there is a lack of consensus regarding this relationship. Thus, this meta-analysis aimed to explore the relationship between smartphone addiction and Eysenck's personality traits in Chinese adolescents, as well as its possible moderators. Through literature search and screening, 33 studies were included, comprising 79 independent effect sizes with a total of 17, 737 subjects. A random effects model was selected, and it was found that smartphone addiction was positively associated with psychoticism (*r* = 0.16, *p* < 0.001) and neuroticism (*r* = 0.32, *p* < 0.001), but not significantly associated with extroversion (*r* = −0.06, *p* = 0.079). The moderating effect test showed that sex and year of study publication had significant influences on the relationship between smartphone addiction and psychoticism, and the year of study publication had a significant influence on the relationship between smartphone addiction and neuroticism. This study is the first meta-analysis on the relationship between smartphone addiction and Eysenck's personality traits among adolescents in China, and the results have helped to clarify the controversy of previous studies regarding this relationship.

## Introduction

According to the 47th Statistical Report on Internet Development released by the China Internet Network Information Center (2021), the number of Chinese mobile Internet users by the end of April 2021 amounted to 940 million. Mobile netizens at this time accounted for 99.2% of the total netizen population, and 10–20 year olds accounted for 20.3%. Smartphones bring numerous benefits to human beings, but they can also lead to smartphone addiction, an trend which has increased year by year, particularly among adolescents. The prevalence of smartphone addiction was found to be at 35% or higher in young smartphone users from different regions across China (Liu Q. X. et al., 2017; Xu and Sun, 2020). The increasing amount of time adolescents spend using smartphones can have many negative physical and mental impacts, such as sleep problems, depression, and poor school performance (Lopez-Fernandez et al., 2014; Gao et al., 2020). As such, smartphone addiction has attracted increasing attention from both government and scholars.

Personality traits, as an important influencing factor of smartphone addiction, have also been a focus of concern (Desola et al., 2016; Zhang et al., 2017). Although there are plenty of studies on the relationship between smartphone addiction and specific personality traits, the findings are mixed (He et al., 2017; Liu Q. X. et al., 2017; Zhang et al., 2018). China as a country has the largest number of smartphone users in the world (Jin, 2018), which does provide researchers with an important data source in regards to analyzing the relationship between smartphone addiction and personality traits among adolescents. However, as far as we know, no meta-analysis has yet been conducted on this topic. Thus, this study aimed to analyze the relationship between smartphone addiction and Eysenck's personality traits among Chinese adolescents through meta-analysis research, and to explore a range of factors that may have contributed to observed inconsistencies in findings by moderating this relationship.

### Concept and Measurement of Smartphone Addiction

Smartphone addiction is characterized by uncontrolled smartphone use that leads to adverse consequences on an individual's physical health, mental health, and social functioning (Billieux et al., 2015). Currently, internationally-used mainstream measuring tools include the Mobile Phone Problem Use Scale (MPPUS) developed by Bianchi and Phillips (2005), the Mobile Phone Addiction Index (MPAI) developed by Leung (2007), and the Smartphone Addiction Scale (SAS) developed by Kwon et al. (2013). In addition to these, Xiong et al. (2012) compiled the Mobile Phone Addiction Tendency Scale (MPATS) using an indigenist research methodology to address and overcome cultural and language issues to measure smartphone addiction in a Chinese context. The MPATS scale has since become widely used in measuring smartphone addiction in adolescents with a high reliability and validity, and the existence of cross-cultural differences can be excluded. There are four dimensions measure by the MPATS: withdrawal symptoms (a series of adverse physiological and psychological reactions when stopping smartphone use), salience (smartphone use occupies a central position in the user's thoughts and behavior), social comfort (the role of the smartphone in interpersonal communication), and mood changes (emotional changes caused by smartphone use).

There also exist some derived scales which are based on the MPATS and combined with results of interviews with respondents (Liu Q. X. et al., 2017). For example, the Smartphone Addiction Scale for College Students (SAS-C) was developed by Su et al. (2014) and includes six dimensions: withdrawal behavior, salience behavior, social comfort, negative effects, use of applications (apps), and renewals of apps. There is also the Smartphone Dependence Scale for College Students (SDS-C), which was developed by Wang et al. (2014) and measures five dimensions: withdrawal, salience, compulsion, tolerance, and harmfulness.

### The Concept and Measurement of Personality Traits

Personality can be defined as a set of psychological qualities related to feelings, thoughts, and behaviors (Eysenck, 1991). Many theories regarding personality structure have been proposed, but the most widely known are the Big-Five Model and Eysenck's Three-Factor Mode. Of these two, Eysenck's Three-Factor Mode was developed earlier and is today the more widely used personality theory (Qian et al., 2000; Yang et al., 2018). It is also the most commonly used theory when evaluating personality traits of smartphone addicts in China. Thus, in this study, we focused on Eysenck's Three-Factor Mode. Eysenck's Three-Factor Mode has three dimensions: extraversion (individuals with high extraversion are more sensitive to external stimulation and more optimistic about their future), psychoticism (individuals with high psychoticism are more hostile toward others and more prone to psychological distress), and neuroticism (individuals with high neuroticism are emotionally unstable; Eysenck, 1991). Prior studies have found that Eysenck's Three-Factor Mode has a substantial empirical foundation (Barrett and Eysenck, 1984; Yang et al., 2018), solid evidence of reliability and construct validity (Yang and Gong, 1994; Qian et al., 2000), and maintains cross-cultural consistency (Bowden et al., 2016). Given that Eysenck's Three-Factor Model is the most widely accepted personality theory used in the Chinese scientific community, the scale was chosen to evaluate the personality traits in this study to perform an in-depth exploration of the relationship between smartphone addiction and Eysenck's personality traits.

### The Relationship Between Smartphone Addiction and Eysenck's Personality Traits

A substantial number of studies have addressed the relationship between smartphone addiction and Eysenck's personality traits in recent years. However, the findings have been mixed, with contradictory results or, at the very least, inconsistency in the relationship strength, direction, and significance. For example, several studies have found that smartphone addiction had a moderately significant negative association with extraversion (Shi et al., 2017; Xu and Wang, 2018; Zhang et al., 2018). This has been framed using social reinforcement theory, which posits that for low extraversion adolescents, smartphone use can effectively enhance their contact with the outside world to make up for their lack of language expression skills and social skills, while simultaneously avoiding embarrassment from to actual interpersonal situation. This indirectly leads to individuals using smartphones more frequently or excessively and increases their risk of smartphone addiction (Bahtiyar, 2015). Meanwhile, others studies have found that extraversion was positively associated with smartphone addiction (Du et al., 2012; Zhang et al., 2016), explained as being due to the difference in individuals' optimal arousal levels. Compared with those with low extraversion, adolescents with high extraversion have high levels of sensation-seeking and need more external stimulation to continuously meet their needs for arousal (Hussain et al., 2017). In terms of stimulus source, the vast amount of information and the rich, novel types of information the smartphone can provide meets those high extraversion stimulation demands.

With regards to the psychoticism dimension, some studies have found that smartphone addiction has a significant positive correlation with psychoticism (Zhang et al., 2016; Zhu and Zhi, 2016). From the perspective of evolutionary psychology, due to the limitations of human ability, people choose to live in groups to make up for their individual shortcomings, and socialization has gradually evolved to become a basic coping style through which individuals can deal with external crises (He et al., 2017). But high psychotic individuals with a low socialization level often have to bear the pressure of life alone due to their lack of effective positive coping strategies. As such, smartphones have gradually become a primary tool for such individuals to find respite from reality (Zhang et al., 2016). However, there have also been studies with opposing findings, that there was no significant correlation between psychoticism and smartphone addiction. For example, Qiu and Zhang (2014) investigated the differences in personality traits of adolescents either with or without smartphone addiction, and found that there was no significant difference in psychoticism between the two groups. Shi et al. (2017) conducted a scale test on 476 college students and also found no significant relationship between psychoticism and smartphone addiction. Consequently, the correlation between smartphone addiction and psychoticism is still uncertain.

Similarly, there is also considerable controversy about the relationship between smartphone addiction and neuroticism (He et al., 2017; Zhang et al., 2018; Xiong, 2019). The majority of studies have based themselves on the theory of emotional dissonance, which suggests that high neurotic individuals are more often in a state of emotional instability, and thus need to seek ways to cope with negative emotions more often (Li et al., 2017; Zhang et al., 2018). Naturally, the convenience and diverse entertainment options of smartphones provide them with more possibilities to regulate their emotions, and the results of these studies suggested a significant positive correlation between smartphone addiction and neuroticism. Nevertheless, there were large differences between these studies, with *r* values ranging from 0.03 to 0.50 (Gao, 2017; Xiong, 2019).

To sum up, as these and other existing studies show mixed results, the relationship between smartphone addiction and Eysenck's personality traits is ambiguous. What is the relationship between smartphone addiction and Eysenck's personality traits, and what are the moderating factors? To determine this, it is necessary to conduct a comprehensive and targeted meta-analysis study of the relevant literature.

### Potential Moderators

It is possible that moderating variables might explain some of the above-mentioned inconsistencies in existing findings regarding smartphone addiction and Eysenck's personality traits. This section describes these potential moderators in detail.

#### Sex

In the field of smartphone addiction, virtually all studies have indicated that females spend significantly more time (Lopez-Fernandez et al., 2012) and money (Roberts et al., 2014) on their smartphones than males, and the proportion of addiction has also been shown to be higher in females (Billieux et al., 2015; Zhang et al., 2019). Meanwhile, there are considerable differences in the content of smartphone use between different the sexes. Some studies have shown that females use them more for voice calls, social networking, and online shopping, while males use them more for watching videos and game applications, and males also demonstrate a higher use risk (e.g., cyber violence) compared to females (Desola et al., 2016). Additionally, differences have been found in personality tendencies between the different sexes, for example, several studies have revealed significant sex differences in extraversion and neuroticism factors. Specifically, compared to males, females score higher on the neuroticism scale and, to a lesser degree, on the extraversion scale (Herrera et al., 2016; Nguyen et al., 2019). Given sex differences in both smartphones use and personality tendencies, then, the relationship between smartphone addiction and Eysenck personality traits could also be moderated by sex.

#### Age

From the perspective of lifelong development, younger ages are associated with having worse self-control. When younger people use a smartphone, it is more difficult for them to resist the temptation of applications such as videos and games, thus increasing their likelihood of developing smartphone addiction (Desola et al., 2016). Several empirical studies have found that the time an individual spends using smartphones declines with age (Desola et al., 2016; Ding and Zhao, 2018; Xiong, 2019). Additionally, Hayes et al. (2015) found that time length, frequency, and intensity of mobile social media use in adolescents were significantly higher than in adults. Therefore, the current study speculated that there might be differences in susceptibility for smartphone addiction among adolescents of different ages, which would affect the relationship between smartphone addiction and Eysenck's personality traits.

#### Year of Publication

Demographic information pertaining to smartphone ownership indicates a steady increase in ownership over the last decade, with the penetration rate of smartphones rising from 74.5 to 99.2% from 2012 to 2021 (China Internet Network Information Center, 2021). With such growth in numbers of users, it is only logical that the problem of smartphone addiction has also become increasingly prominent. A previous meta-analysis on behavioral addiction showed that the year of publication plays an important moderating role in findings (Gao et al., 2020; Pan et al., 2020). Therefore, the current study speculated that year of publication might moderate the relationship between smartphone addiction and Eysenck's personality traits.

#### Measuring Tools

Currently, there is lack of uniform regulations or evaluation standards regarding the measurement tools for smartphone addiction. The different tools have differences in dimension division, item numbering, and scoring methods, all of which may affect study results. Eisenberg and Miller (1987) pointed out that differences in measurement tools can directly affect the strength of the relationship between different variables. Therefore, the relationship between smartphone addiction and Eysenck's personality traits could be affected by differences between measurement tools used to evaluate smartphone addiction. Finally, in addition to the possible moderators already mentioned in this section, the current study also attempted to explore the potential impacts of certain demographic variables that have been ignored in the past, such as sample region, with a view to fill these voids in the existing empirical research.

### Purpose of This Study

Great differences exist between the results of different studies despite the fact that substantial empirical research has already been accumulated on the relationship between smartphone addiction and Eysenck's personality traits, which is vital for researchers to conduct accurate quantitative analysis. A meta-analysis (quantitative systematic review) is a statistical technique for amalgamating, summarizing and reviewing primary quantitative research. By combining information from all relevant studies, meta-analyses can provide more accurate estimates than those derived from the individual studies included within a review (Glass, 1976). Two previous studies have tried to conduct meta-analyses on the association between smartphone addiction and personality traits, but one of these studies included only 16 articles, which might not effectively represent the actual levels of smartphone addiction and Eysenck's personality traits. Moreover, the samples used were college students only, and the moderator only examined the measurement tools and literature types, but did not analyze the reasons for the inconsistencies between previous study results (Guo, 2017). The second existing meta-analysis investigated the relationship between smartphone addiction and the Big Five personality traits rather than Eysenck's personality traits, and the samples were not focused on the adolescent population in China (Gao et al., 2020). This study therefore aims to clarify the utility of the relationship between smartphone addiction and Eysenck's personality traits in consideration of Chinese adolescents by using meta-analysis techniques, while also investigating a range of factors that may have contributed to the observed inconsistencies between previous findings by moderating this relationship. The intent of the current study is to clarify the controversy in existing research conclusions, and to provide explanations and evidence from a Chinese adolescent population regarding the relationship between smartphone addiction and Eysenck's personality traits.

## Methods

### Literature Search

Due to the fact that the smartphone was only popularized in Chinese mainland in 2012 (Xie, 2013), the study publishing dates included in this meta-analysis ranged from 2012 to 2021 to eliminate interference caused by studies looking at non-smartphone mobile phones. We searched for empirical studies focused on the relationship between smartphone addiction and Eysenck's personality traits published between January 2012 and June 2021. Chinese databases used included: China National Knowledge Infrastructure (CNKI), Chongqing VIP Information Co., Ltd (CQVIP), and Wan-Fang DATA. English databases used included: EBSCO, Web of Science, Psy INFO, and Google Scholar. The following keywords were used: for smartphone addiction, “smartphone addiction,” “mobile phone addiction,” and “mobile phone dependence;” for Eysenck's personality traits, “Eysenck personality,” “neuroticism,” “extraversion,” and “psychoticism.” To avoid unintentional omissions, we then conducted a “backward search,” where the reference sections of the studies found in the initial searches were used to find other relevant articles. Each article was screened according to the following criteria: (1) It must have reported on quantitative research between smartphone addiction and at least one dimension of Eysenck's personality traits; (2) The study reported sufficient statistical detail (e.g., *r, t, F*, and χ^2^) to allow the calculation of correlations between smartphone addiction and Eysenck's personality traits; (3) All studies must have reported sample size, and each sample must have been independent, excluding cross-samples or duplicate publications; (4) The sample used is made up of Chinese adolescents, excluding mentally ill, left-behind adolescents, or other special individuals; (5) Low quality literature was not included. The current meta-analysis was conducted in accordance with the Preferred Reporting Items for Systematic Reviews (PRISMA), which provides detailed guidelines describing the methods involving eligibility criteria, information sources, search strategy, etc. for performing a meta-analysis (Moher et al., 2009).

### Literature Quality Assessment and Coding

Two reviewers independently assessed the quality of each of the studies using an assessment tool for cross-sectional studies that applies to correlational study design (Ivie et al., 2020). If there was a dispute in the process of the screening an article, the two reviewers would review the article together and differences of opinion would be discussed until an agreement was found. The quality assessment tool consisted of nine items which were each rated as poor (0), fair (1), or good (2) by each independent reviewer. The final score for each article ranged from 0 to 18. An article with a final score of 1–5 was considered to be of low quality, 6–13 was considered to be of moderate quality, and 14–18 was considered to be of high quality. Agreement between the two reviewers' evaluations was determined using a kappa value, with a kappa value of < 0.40 indicating weak consistency, from <0.40 to <0.70 indicating common consistency, and > 0.75 indicating high consistency (Orwin, 1994). The current study showed a high kappa value at 0.87.

The collected articles were coded as follows: first author, year of publication, sex ratio (female), sample size, average age, sample region (i.e., Eastern China, Northeastern China, Central China, and Western China), and the scale used to measure smartphone addiction. Effect size was calculated based on each independent sample only once. If an article included multiple independent sample sizes or outcome indicators, these were divided into a multiple effect size estimates. If sample sizes or outcome indicators were not reported in the article, or if it was not clear about most of the moderated variables in the study, the article would be excluded in order to maintain accuracy of results in the current study.

The coding process had three phases. First, the corresponding author referred to the article data and encoded the article information. Second, based on the inclusion criteria, the primary studies were independently coded by two of the authors of this current study. Finally, the two coders cross-checked the coding results to verified the accuracy of the data. The comparisons in this final step showed high consistency (98%). The Appendix presents the details of all studies included in this meta-analysis.

### Computation of Effect Sizes

The correlation coefficient *r* evaluates the relationship between smartphone addiction and Eysenck's personality traits. Some articles did not report the correlation coefficient but did report *t, F*, and χ^2^, which we converted into a correlation coefficient based on the corresponding formula, namely *r* = [*t*^2^/(*t*^2^ + *df* )]^1/2^, *df* = *n*_1_ + *n*_2_ − 2; *r* = [*F*/(*F* + *df* )]^1/2^, *df* = n_1_ + *n*_2_ − 2; *r* = [χ^2^/(χ^2^ + *N*)]^1/2^ (Rosenthal, 1991; Lipsey and Wilson, 2001; Zheng et al., 2011). After obtaining the original correlation coefficient *r* between smartphone addiction and Eysenck's personality traits, all correlation coefficients were transformed into Fisher's *z*-values to avoid the problematic standard error formulation of the *r*-values. Next, the Fisher's *z*-values and confidence intervals were converted back to correlation coefficients to make their interpretation easier (Lipsey and Wilson, 2001).

### Data Processing and Analysis

The statistical analysis was conducted using comprehensive meta-analysis software (CMA Version 2.0), which is a program developed specifically for use in meta-analysis. It mainly includes three modules—Data entry, Data Analysis, and High resolution plots. The specific operation method of CMA 2.0 is detailed in the website https://www.meta-analysis.com. Heterogeneity tests were used to determine whether each result was significantly different from the overall effect size. Heterogeneity among effect sizes were determined by computing a *Q* statistic (Lipsey and Wilson, 2001), and effect sizes were determined to be heterogenous when the *Q* statistic was significant. Statistical heterogeneity was also assessed using *I*^2^ statistic (Huedo-Medina et al., 2006); a value of 25, 50, or 75% was considered as having low, moderate, or high levels of heterogeneity, respectively. If heterogeneity was high between studies, a random-effects model was used; if instead there was homogeneity, a fixed-effects model was used (Berkeljon and Baldwin, 2009). To further analyze the origin of heterogeneity, moderator variables that contribute to heterogeneity were looked for using the subgroup analysis (categorical variables) and meta-regression analyses (continuous variables; Huedo-Medina et al., 2006).

Publication bias refers to the potential issue that true or full results may not include in a study due to the fear of it not being published if the results lack strength, resulting in a lack of representativeness of the sample or the article not including statistically non-significant findings. The current study employed a funnel plot, Rosenthal's fail-safe N (*N*_*fs*_), and Egger's regression test to assess publication bias. First, if the effect sizes (small circles) observed in the funnel plot were distributed symmetrically around the vertical line, this suggested that potential publication bias was negligible (Viechtbauer, 2007). Second, Rosenthal's fail-safe N (*N*_*fs*_) was used to report a significance level based on *k* studies, with larger *N*_*fs*_ meaning that results were less prone to publication bias. An *N*_*fs*_ of > 5*k* + 10 (*k* is the number of studies included) suggested no publication bias (Borenstein et al., 2009). Finally, using the Egger's regression test, and if the results of the Egger's intercept were not significant, it was determined that publication bias did not exist.

## Results

### Results of the Literature Search

**Figure 1:**
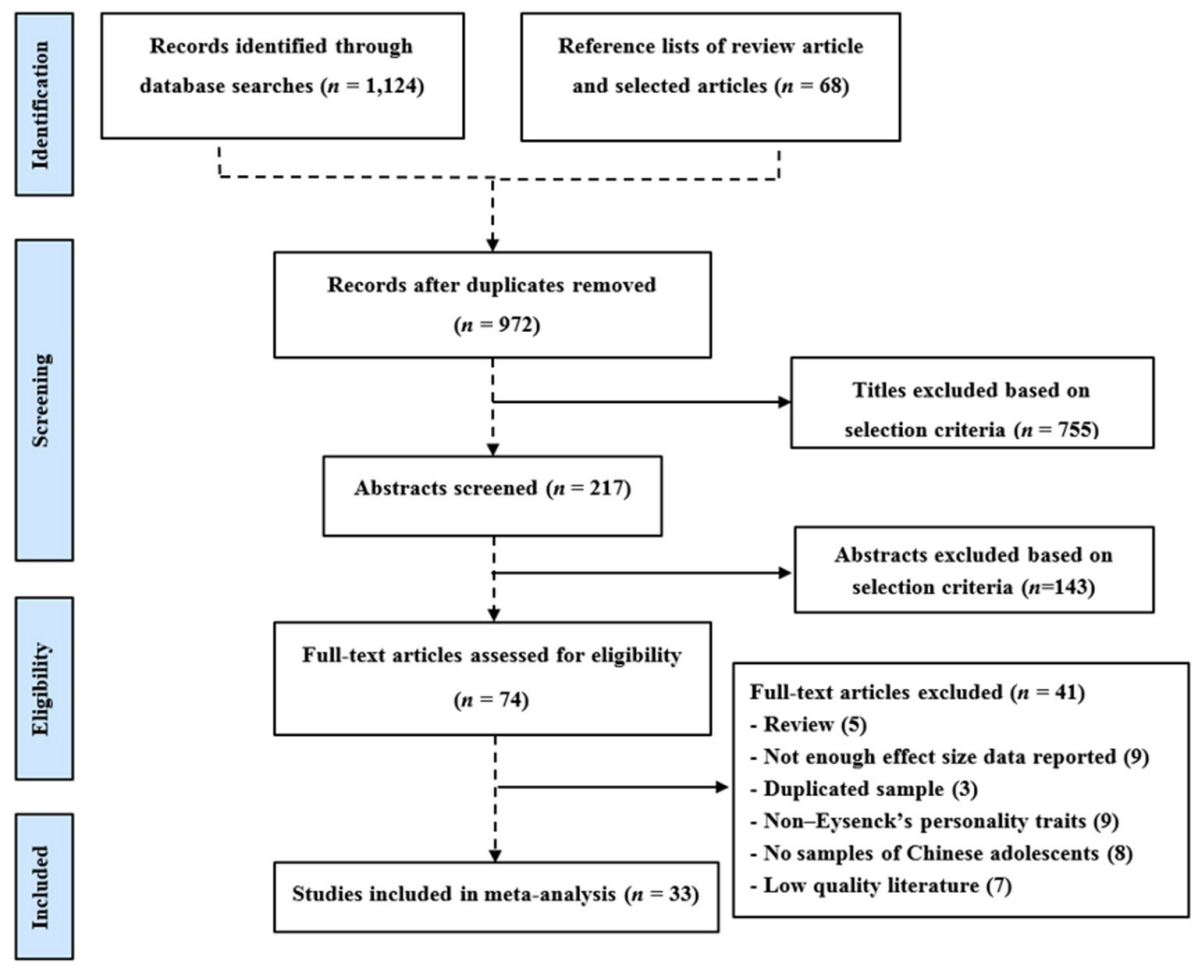
Flowchart describing study selection process.

A flowchart that visually depicts the selection procedure is provided in Figure 1. In total, 33 studies (*N* = 17,737) yielding 79 independent samples were included in the current meta-analysis, based on the previously outlined selection criteria. Among them were two English publications (Chinese mainland sample) and 31 Chinese publications. Sample sizes ranged 168–2,092 individuals. Participants were from the Eastern, Northeastern, Western, and Central regions of China, covering a total of 15 provincial administrative regions. Sample participants were all Chinese mainland adolescents with sample age means ranging from 16.00 to 21.43 years. The quality assessment scores of all the studies ranged from 11 to 18 with an average score of 15.11, and the overall quality was relatively good.

All studies used self-report scales to measure both smartphone addiction and Eysenck's personality traits. Of the 33 studies, 22 studies used Mobile Phone Addiction Tendency Scale (MPATS) to assess mobile phone addiction in Chinese adolescents. The remaining studies used a variety of other measures such as the Smartphone Addiction Scale for College Students (SAS-C) and the Smartphone Dependence Scale for College Students (SDS-C). According to the suggestion of Card (2012), statistical differences between subgroups were only assessed when subgroups contained at least six independent studies per subgroup. As a consequence, the number of literatures using the SAS-C and SDS-C as measurement tools was too small to calculate the individual effects of these measures. Given the effectiveness and comprehensiveness of the study, SAS-C and SDS-C were referred to collectively as others.

### Effect Size and Homogeneity Tests

As shown in Table 1, the results of the *Q* statistic for heterogeneity were statistically significant for each trait, and all *I*^2^ were over 75%, suggesting that the observed dispersion of effect sizes was mostly due to true heterogeneity, which therefore warranted the use of the random effect model (Berkeljon and Baldwin, 2009). Lipsey and Wilson (2001) have suggested that an effect size of *r* ≤ 0.10 can be regarded as having a low correlation, while <0.10 *r* < 0.40 and *r* ≥ 0.40 can be regarded as having moderate and high correlations, respectively. The average cumulative effect size in the current study indicated that extraversion (*r* = −0.06, *p* = 0.079) was not significantly associated with smartphone addiction, but that psychoticism (*r* = 0.16, *p* < 0.001) and neuroticism (*r* = 0.32, *p* < 0.001) were moderately positively associated with smartphone addiction.

**Table 1:** Heterogeneity of effect sizes and publication bias.

**Outcome measures**	***k***	***N***	***R***	**Heterogeneity tests**			**Publication bias**			
				***Q***	***Df***	***I^**2**^***	***N_***fs***_***	**Egger's intercept**	***SE***	**95%CI**
Extraversion	23	13,261	−0.06	407.32^***^	22	94.59	504	6.25	3.03	[−0.05, 12.56]
Psychoticism	25	12,281	0.16^***^	200.11^***^	24	88.01	1,931	−1.63	1.96	[−5.67, 2.41]
Neuroticism	31	16,980	0.32^***^	242.54^***^	30	87.63	4,220	−2.84	1.79	[−6.50, 0.81]

*k, number of effect sizes; N, Sample size*.

^*^p < 0.05, ^**^p < 0.01,

*** *p < 0.001*.

### Publication Bias

**Figure 2:**
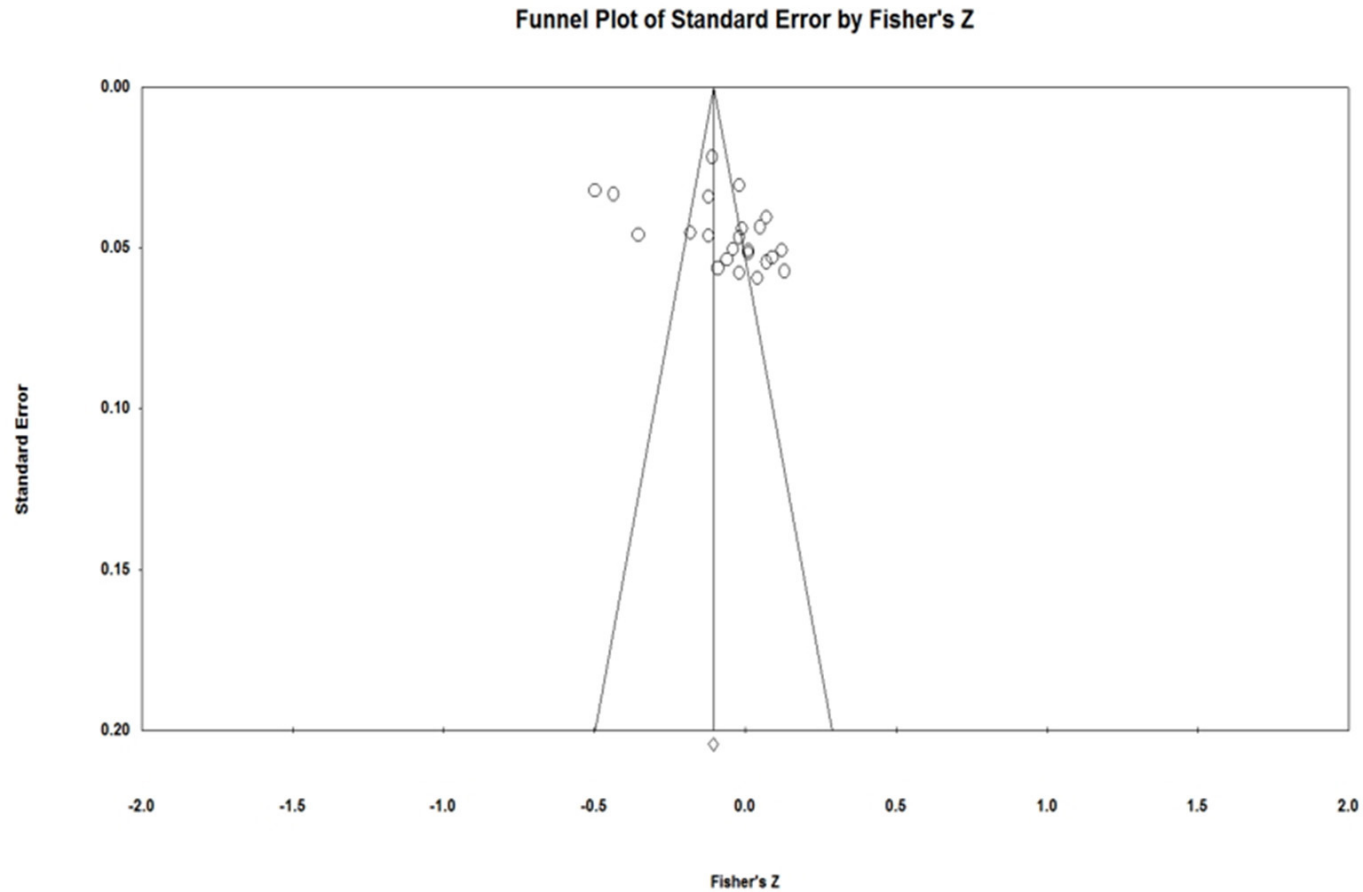
Funnel plot for Eysenck's extraversion.

**Figure 3:**
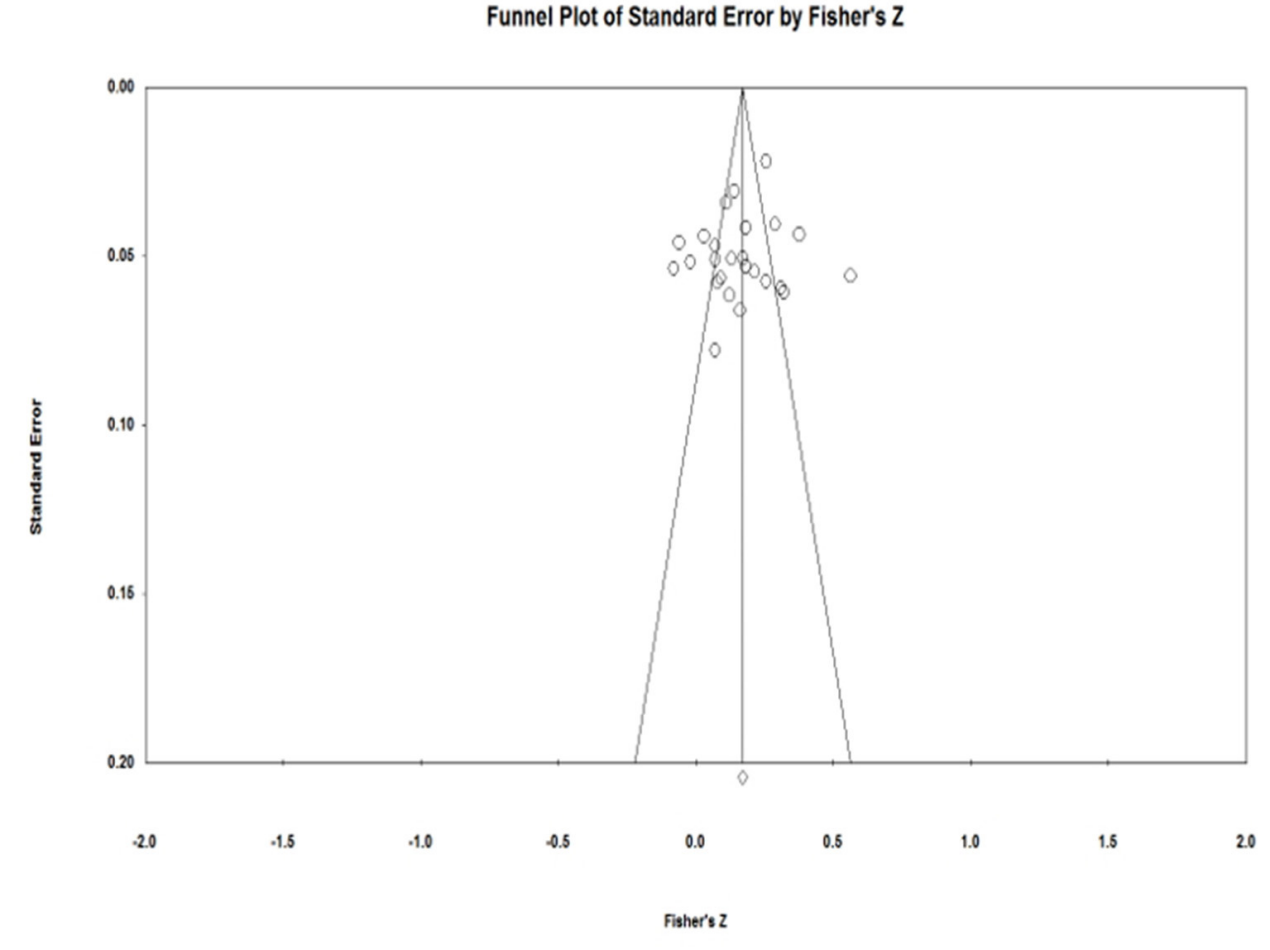
Funnel plot for Eysenck's psychoticism.

**Figure 4:**
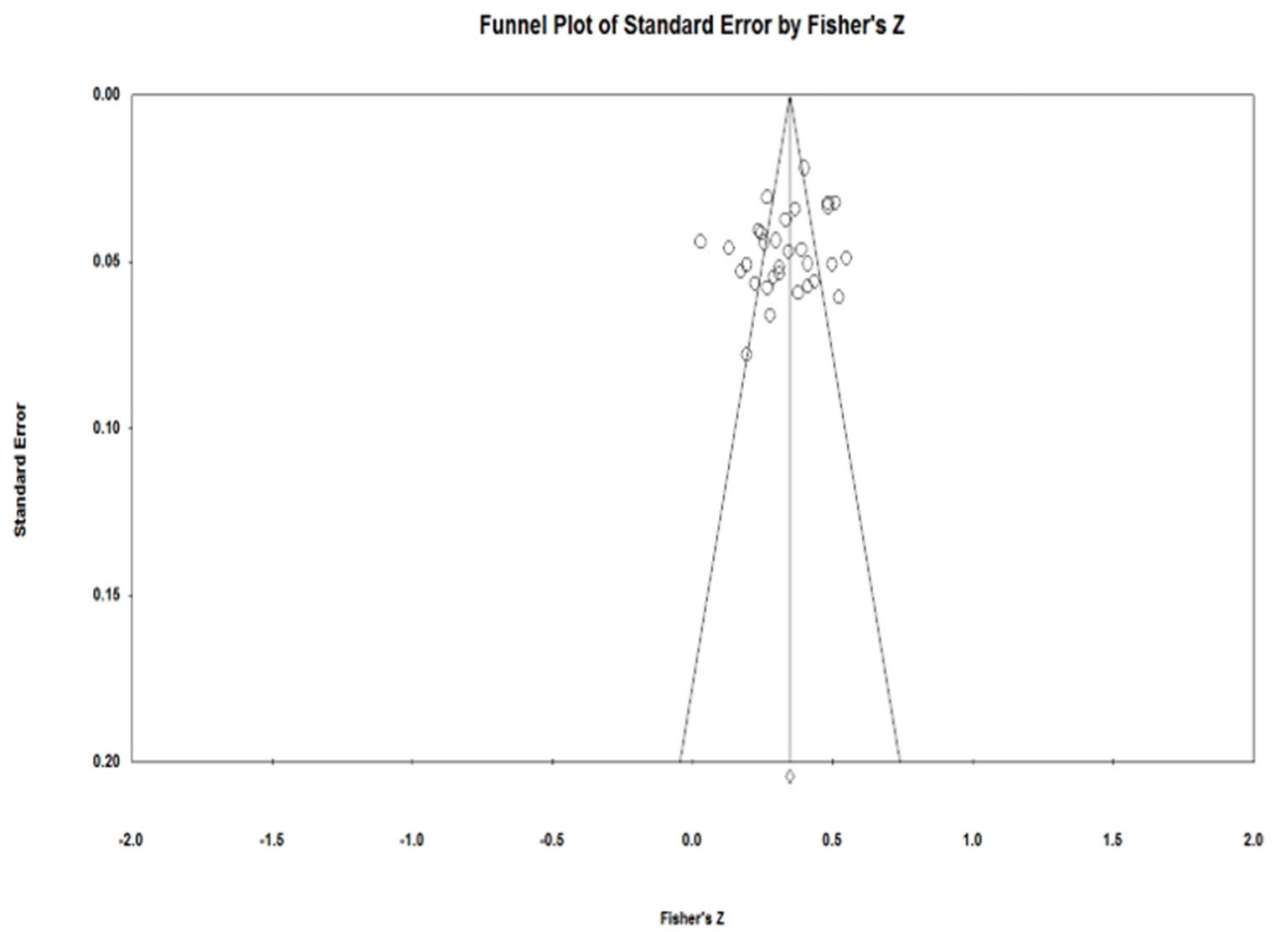
Funnel plot for Eysenck's neuroticism.

The funnel plots (Figures 2–4) of each of the Eysenck's personality traits were nearly symmetrical, which can be understood as indicating that there was no publication bias (Viechtbauer, 2007). Given the subjectivity involved in interpreting a funnel plot, however, we used Rosenthal's fail-safe N (*N*_*fs*_) as well as Egger's regression test to further assess publication bias (Egger and Smith, 1998; Borenstein et al., 2009). As Table 1 shows, the *N*_*fs*_ of extraversion, psychoticism, and neuroticism was calculated to be 504, 1,931, and 4,220, respectively, and each of these were larger than the criteria of 5*k* + 10, which suggests no presence of publication bias. Similarly, Egger's regression test showed that the 95% confidence interval for the intercept of each of Eysenck's personality traits included zero, further indicating that the studies included in the meta-analysis did not show significant publication bias.

### Sensitivity Analysis

It is important to keep in mind that outlying literature could disturb the results of a meta-analysis (Lipsey and Wilson, 2001). As such, sensitivity analyses were used to evaluate the robustness of the studies' research results by calculating how the average cumulative effect size changed by removing one study at a time (Borenstein et al., 2009; Li et al., 2019). The results showed that in the relationship between smartphone addiction and each of Eysenck's personality traits, the significance level of average cumulative effect size of deleting any one piece of literature had no change. In performing this analysis, the maximum change in effect size of extraversion was 0.014, the maximum change of effect size of both psychoticism and neuroticism was 0.010. The absolute difference of average cumulative effect size was <20% both before and after eliminating any data, indicating that the meta-analysis results were quite robust (Field et al., 2020).

### Moderation Analyses

The results thus far showed significantly large heterogeneity in the effect size, which suggested the need for exploring potential moderators. Given that the effect size of smartphone addiction and extroversion are not significant, it was of no practical significance to analyze the moderating effects (Lu and Hang, 2007). Consequently, the current study instead explored whether potential moderators affected the relationships between smartphone addiction and both psychoticism and neuroticism, as shown above in Tables 2, 3.

**Table 2:** Results of categorical and continuous moderators for the association between smartphone addiction and psychoticism.

**Moderators**		***k***	***N***	***r/*slope**	**95% CI**	**Heterogeneity *Q***
Region	Eastern	6	3,548	0.19	[0.13, 0.26]	1.62
	Northeastern	4	1,777	0.14	[−0.01, 0.28]	
	Central	9	4,059	0.13	[0.04, 0.21]	
	Western	3	1,231	0.21	[−0.10, 0.49]	
Measurement tools	MPATS	19	10,543	0.15	[0.10, 0.20]	0.29
	Others	6	1,738	0.19	[0.04, 0.34]	
Year of publication		25	12,281	−0.01	[−0.02, −0.01]	8.02^**^
Mean age		8	5,108	−0.01	[−0.02, 0.02]	0.01
Sex (% female)		22	11,172	−0.31	[−0.49, −0.13]	11.06^***^

r, effect sizes of categorical variables; slope, effect sizes of continuous variable. ^*^p < 0.05,

** *p < 0.01*,

*** *p < 0.001*.

**Table 3:** Results of categorical and continuous moderators for the association between smartphone addiction and neuroticism.

**Moderators**		***k***	***N***	***r/*slope**	**95% CI**	**Heterogeneity *Q***
Region	Eastern	8	4,965	0.30	[0.23, 0.37]	2.42
	Northeastern	6	3,467	0.31	[0.22, 0.39]	
	Central	10	4,685	0.35	[0.29, 0.41]	
	Western	3	1,231	0.21	[−0.01, 0.42]	
Measurement tools	MPATS	21	11,980	0.31	[0.27, 0.36]	0.11
	Others	9	4,278	0.33	[0.24, 0.41]	
Year of publication		31	16,980	−0.01	[−0.01, 0.01]	0.05
Mean age		12	8,390	0.02	[−0.01, 0.03]	2.72
Sex (% female)		28	15,871	−0.25	[−0.39, −0.11]	12.01^***^

^*^p < 0.05, ^**^p < 0.01,

*** *p < 0.001*.

Subgroup and meta-regression analyses showed that sex had a negative moderating effect on the relationships between smartphone addiction and both psychoticism and neuroticism which indicated that, as the number of females in a sample increased, the relationships between smartphone addiction and both psychoticism and neuroticism gradually decreased. Year of publication was found to have a negative moderating effect on the relationship between smartphone addiction and psychoticism, which indicated that as the publication year became more recent, the relationship between smartphone addiction and psychoticism gradually decreased. The remaining hypothesized moderators did not account for significant heterogeneity.

## Discussion

The current meta-analysis aimed to determine the relationship between smartphone addiction and Eysenck's personality traits in Chinese adolescents and evaluate possible moderators of this relationship. The results show that smartphone addiction has a positive association with both psychoticism and neuroticism, but no association with extraversion. In addition, the data revealed that sex and year of study publication had significant influences on the relationship between smartphone addiction and psychoticism, and the year of study publication had a significant influence on the relationship between smartphone addiction and neuroticism.

### The Relationship Between Smartphone Addiction and Eysenck's Personality Traits

This study revealed that smartphone addiction has a non-significant correlation with extraversion in Chinese adolescents (*r* = −0.06, *p* = 0.079). This illustrates that following the growth in popularity of smartphones in China, and their continuous development toward the current 5G (fifth-generation), the unique draw as well as practicality of smartphones has been highly attractive to Chinese adolescents, both introverted and extroverted. Low extraversion adolescents can effectively enhance their social skills and improve their interpersonal environment through frequent use of smartphones, while adolescents with higher extraversion find that smartphones can meet their needs for increased external stimulation and improve their levels of arousal *via* access to massive amounts of information and tempting application software (Bahtiyar, 2015; Hussain et al., 2017). As a result, there appears to be little difference in the risk of smartphone addiction between introverted and extroverted Chinese adolescents. An additional point worth noting is that this result is inconsistent with the meta-analysis conclusion of Gao et al. (2020), which reported a low correlation between smartphone addiction and extraversion (*r* = 0.07, *p* < 0.05). Two interpretations can be made of these findings. First, that in the current study, the subjects in focus are Chinese adolescents, while Gao et al.' s research subjects came from multiple countries and were primarily adults. Second, although Gao et al. (2020) found that extraversion was significantly positively correlated with smartphone addiction, the effect size is small, reflecting that the relationship between smartphone addiction and extraversion is also weak if it even exists.

Consistent with the perspective of evolutionary psychology, the current study found that there was a moderate positive correlation between smartphone addiction and psychoticism (*r* = 0.16, *p* < 0.001). As a basic human need, socialization is also a primary coping tactic in helping individuals deal with external crises (He et al., 2017). Compared to those with low psychoticism, individuals with high psychoticism generally have more hostility toward others, which in turn causes them to suffer from isolation (Dunlop et al., 2012). Consequently, individuals with high psychoticism find it difficult to integrate into their surrounding environment due to their lack of effective positive coping strategies. This further deepens their antisocial tendencies, and can lead them to vent their resentment through violent or destructive behaviors when confronted with external conflict (Wang et al., 2014). Such behavior does generate a higher risk of social cost in regular society due to the existence of laws, regulations, and the consequential punishment given for breaking these, however by making use of cyber aggression, these individuals with high psychoticism can act on their impulses with a relatively reduced social risk cost (He et al., 2017). Therefore, violent videos, graphic games, and horror movies on the Internet have become popular ways for such individuals to vent themselves (Zhang et al., 2016), which in turn increases their frequency of smartphone and other network media usage.

The current study also found a moderate positive correlation between smartphone addiction and neuroticism (*r* = 0.32, *p* < 0.001), which supports the theory of emotional dissonance (Li et al., 2017). This suggests that individuals high in neuroticism lack emotional regulation abilities and consequently suffer from frequent emotional fluctuations. For this reason, the virtual world provided by smartphones can provide them with comfort from these stressful emotions (Gao et al., 2017). Particularly in regards to education, Chinese parents attach great importance to their adolescents' academic performance as the parents generally believe that education is their child's best way to success, and Chinese traditional culture encourages adolescents to strive to achieve good grades, for themselves but also for their parents. Thus, Chinese adolescents usually face enormous academic pressure and an excessive amount of homework, the stress of which makes them more vulnerable to extreme emotions (Edward et al., 2017). Furthermore, adolescents' abilities in self-management and willpower are generally weak at this point in their development, and smartphones are portable, extensive, and covert, particularly when compared with other types of media or devices. This could explain why smartphone addiction and neuroticism are more closely connected in Chinese adolescents (Xiong, 2019).

### Significant Moderators

As the strength of the relationship between smartphone addiction and Eysenck's personality traits may differ between the various sexes, measurement tools, ages, publication years, and regions, one single study cannot accurately reflect the accurate strength of this relationship. Therefore, the current study explored the possible moderators that could affect this relationship through subgroup and meta-regression analyses.

With regards to moderation due to sex, the current study found that the proportion of females in a sample significantly negatively moderated the relationship between smartphone addiction and psychoticism, which indicates that this relationship was found to be stronger in samples containing more males. This is in line with previous findings which have suggested that variation exists between sexes due to differences in their use of smartphone content, as well as the fact that females have been shown to prioritize social interaction and online shopping in their smartphone use while males are more likely to play games and watch videos (Desola et al., 2016). Meanwhile, males are exposed to more violent games or videos and are thus more likely to exhibit higher levels of aggressive behavior than females (Vangeel et al., 2017). Therefore, males with high psychoticism are also more likely to relieve their stress through playing violent games than females with high psychoticism, thus increasing the possibility of smartphone addiction and resulting in a stronger relationship between smartphone addiction and psychoticism in males than females. Similarly, the current study also found that females had a lower correlation between smartphone addiction and neuroticism than males. It is possible that females' emotional intelligence level and emotion regulation strategies are generally better than males' (Zhang et al., 2018). As a result, females would have more ways to regulate their emotions (e.g., talking to people, crying, etc.) rather than simply turning to their smartphones when experiencing mood fluctuations.

Regarding the year of publication, this meta-analysis included only papers published from 2012 to 2021, and found that the year of publication significantly negatively moderated the relationship between smartphone addiction and psychoticism. In other words, this positive relationship weakened with the increase of the year of publication. In recent years, the Chinese government has developed and refined what's termed the “Clean Network,” where administrative organs in the areas of cyberspace management were ordered to step up efforts to restrict or limit online access to violent games and videos and other information perceived as harmful, resulting in a gradual decrease in the probability of individuals coming in contact with violent videos and games while using the Internet (Sun, 2016). Therefore, this Clean Network has likely reduced the frequency of high psychotic individuals being able to access violent games or videos through smartphone to a certain extent. At the same time, this study also found that the relationship between smartphone addiction and neuroticism did not change significantly with the year of publication. This illustrates that the smartphone, as a primary tool of today's society, can be used to adjust or manage personal emotions or pressures, but can also easily lead to smartphone dependency, particularly in individuals with emotional instability, and that this state then remains unchanged over time (Billieux et al., 2015).

### Non-significant Moderators

Contrary to expectations, the relationships between smartphone addiction and both psychoticism and neuroticism were not shown to be moderated by region, suggesting that these relationships remains similar across different regions in China. This study divided the Chinese mainland into four regions based on levels of economic development. The economic level in the Eastern China is the highest, followed by Central and Northeastern China, with Western China as the lowest (Wang and Jin, 2002). The moderation effect of regional economies is not significant on the relationship between smartphone addiction and either psychoticism or neuroticism, which may be because smartphones have essentially achieved universal coverage across the Chinese mainland, and most smartphone applications in China today, such as WeChat and QQ, are free of charge or low cost. As a result of such accessibility, then, regional economic level as a moderating variable is reduced.

Inconsistent with most prior studies, certain studies have investigated whether smartphone addiction tends to decrease with the age of individuals (Desola et al., 2016; Hwang and Park, 2017; Ding and Zhao, 2018). The current study found that the relationships between smartphone addiction and both psychoticism and neuroticism were not moderated by an individual's age, which may be because previous studies have been mainly focused on whole age ranges of individual development (Desola et al., 2016; Liu Q. X. et al., 2017). In the current study, the sample in focus was only adolescents, which suggests that the relationships between smartphone addiction and both psychoticism and neuroticism may have stage stability in adolescence. Meanwhile, it should also be noted that the age range of samples included in this study is 16.00–21.43 years, which does not encompass the whole stage of adolescence. In the future, this age range focus should be further expanded to verify whether the conclusion is robust.

Further, smartphone measurement tools were not found to be a significant moderator of the relationships between smartphone addiction and psychoticism or neuroticism, which reflects the high consistency between smartphone measurement tools. It could be that, despite the differences between MPATS and other tools in terms of item numbers, dimensions, or scoring methods, the theoretical basis of each of the smartphone measurement tools is based on the Internet addiction standard as established by Young (2009). Consequently, differences between different smartphone addiction measurement tools used in a Chinese context are more reflected in the form, while the actual test content may not be different (Liu Q. X. et al., 2017). There is a strong consistency between them, which eliminated the concern regarding interference of measurement tools on the results of the current study.

## Limitations And Future Directions

This study is the first meta-analysis on the relationship between smartphone addiction and Eysenck's personality traits among adolescents in China. The results have helped to clarify the controversy of previous studies regarding this relationship, and have identified potential moderators affecting this relationship. We believe that the focus on Chinese adolescents also makes a special and important contribution to the existing literature. However, some limitations should be noted: (1) This study investigated the relationship between smartphone addiction and Eysenck's personality traits in Chinese adolescents, but failed to take into account all personality types, such as those included in the Big Five Personality framework. Future research should further expand its scope to comprehensively understand the relationship between smartphone addiction and a wider range of personality dimensions of Chinese adolescents. (2) In the subgroup analysis, the distribution of the number of regulatory variables was not balanced, and some sample sizes were too small, which may have affected the research results. (3) Few longitudinal studies were available to evaluate changes in the relationship between smartphone addiction and Eysenck's personality traits over time. (4) Finally, as not all the primary studies provided sufficient data points and information from reviewed studies, we were unable to test potential moderators such as rural/urban areas or educational attainment. We think that these moderators are important for the relationship between smartphone addiction and Eysenck's personality traits in the context of Chinese society and, as such, warrant further investigation.

## Data Availability Statement

The raw data supporting the conclusions of this article will be made available by the authors, without undue reservation.

## Author Contributions

SX started the original study conceptualization and drafted the Introduction and Discussion sections. YX, LZ, and JX conducted data collection, quality assessment, and coding of the studies. BZ conducted the data analysis and made a draft for the Results and Methodology section. All authors read and approved the manuscript. All authors contributed to the article and approved the submitted version.

## Funding

This study was supported by the project of the National Education Scientific Planning, Youth Foundation of Ministry of Education: a study on the heterogeneous developmental trajectory and precise collaborative governance of mobile phone addiction among adolescents from the perspective of cumulative ecological risk [Project No. EBA210397].

## Conflict of Interest

The authors declare that the research was conducted in the absence of any commercial or financial relationships that could be construed as a potential conflict of interest.

## Publisher's Note

All claims expressed in this article are solely those of the authors and do not necessarily represent those of their affiliated organizations, or those of the publisher, the editors and the reviewers. Any product that may be evaluated in this article, or claim that may be made by its manufacturer, is not guaranteed or endorsed by the publisher.
