# Supplementary material for: Smartphone Addiction and Eysenck's Personality Traits Among Chinese Adolescents: A Meta-Analysis
Source: Front Psychol. 2022 Feb 4;12:794112. doi: 10.3389/fpsyg.2021.794112 (PMC8854182; doi:10.3389/fpsyg.2021.794112)
Supplement: Supplementary file 1 [file Table_1.docx]

**Appendix**

Characteristics of Primary Studies Included in the Meta-analysis

| Study (year) | Sample size | Female% | age | age range | Region | Measurement tools | Personality dimension | *r* |
| --- | --- | --- | --- | --- | --- | --- | --- | --- |
| Xiao, 2012 | 529 | 0.44 | – | – | Central | MPATS | E & P & N | 0.05 & 0.36 & 0.29 |
| Liu, 2014 | 392 | 0.67 | – | – | Central | MPATS | E & P & N | 0.01 & 0.13 & 0.46 |
| Zhang, 2017 | 459 | 0.59 | 16.82 | – | Central | MPATS | E & P & N | -0.02 & 0.07 & 0.33 |
| Xiong, 2019 | 421 | 0.71 | 20.28 | 15–22 | Central | Others | N | 0.50 |
| Zeng, 2018 | 395 | 0.59 | – | – | Central | MPATS | E & P & N | -0.04 & 0.17 & 0.39 |
| Chi & Sheng, 2019 | 968 | 0.39 | 21.43 | – | Northeastern | Others | E & N | -0.46 & 0.45 |
| Du & Liang, 2012 | 390 | 0.54 | – | – | Western | Others | E & P & N | 0.12 & 0.07 & 0.19 |
| Shi et al., 2017 | 476 | 0.62 | – | 17–24 | Central | MPATS | E & P & N | -0.34 & -0.06 & 0.13 |
| Xu et al., 2017 | 586 | 0.68 | 20.62 | 18–25 | Northeastern | MPATS | P & N | 0.18 & 0.24 |
| Gao, 2017 | 518 | – | – | – | Western | MPATS | E & P & N | -0.01 & 0.03 & 0.03 |
| Zhang & Liang, 2020 | 859 | 0.59 | 20.27 | 17­–24 | Central | MPATS | E & P & N | -0.12 & 0.11 & 0.35 |
| Wu & Du, 2015 | 340 | 0.72 | – | – | Eastern | MPATS | E & P & N | 0.07 & 0.21 & 0.28 |
| Wang, 2012 | 287 | 0.60 | – | – | Eastern | MPATS | E & P & N | 0.04 & 0.30 & 0.36 |
| Zhang et al., 2015 | 307 | 0.69 | – | – | Central | MPATS | E & P & N | 0.13 & 0.25 & 0.39 |
| Qiu et al., 2014 | 376 | 0.60 | 19.40 | 17­–24 | Central | MPATS | E & P & N | 0.01 & -0.02 & 0.30 |
| Li & Wang, 2014 | 232 | – | – | – | Northeastern | Others | P & N | 0.16 & 0.27 |
| Ge et al., 2013 | 323 | 0.50 | – | – | Western | Others | P & N | 0.51 & 0.41 |
| Guo & Fang, 2017 | 317 | 0.66 | – | – | – | MPATS | E & P & N | -0.09 & 0.09 & 0.22 |
| Hong & Xiao, 2013 | 266 | 0.57 | 20.12 | – | Central | Others | P | 0.12 |
| Xu & Wang, 2018 | 491 | 0.43 | 19.71 | – | Central | MPATS | E | -0.18 |
| Zhang et al., 2016 | 276 | 0.70 | – | – | – | MPATS | P & N | 0.31 & 0.48 |
| Zhang et al., 2016 | 901 | 0.45 | – | – | Eastern | Others | E & N | -0.41 & 0.45 |
| Liang et al., 2016 | 966 | 0.57 | 19.30 | 18–22 | – | MPATS | N | 0.47 |
| Zhu & Liu, 2016 | 2092 | 0.41 | 18.82 | 16–23 | Eastern | MPATS | E & P & N | -0.11 & 0.25 & 0.38 |
| Gan & Ding, 2015 | 302 | 0.46 | 20.15 | – | Eastern | MPATS | E & P & N | -0.02 & 0.14 & 0.26 |
| Wu, 2015 | 168 | 0.55 | 16.00 | – | Eastern | Others | P & N | 0.07 & 0.19 |
| Cheng & Jin, 2018 | 1073 | 0.66 | – | – | – | MPATS | E & P & N | -0.02 & 0.14 & 0.26 |
| Zhu et al., 2017 | 516 | 0.54 | – | – | Eastern | Others | N | 0.25 |
| He & Xia, 2020 | 471 | 0.75 | 20.43 | 18–24 | Central | MPATS | E & N | -0.12 & 0.37 |
| Zhao, 2016 | 350 | 0.47 | – | – | Northeastern | MPATS | E & N | -0.06 & -0.08 & 0.30 |
| Wong, 2019 | 359 | – | – | – | Eastern | Others | E & P & N | 0.09 & 0.18 & 0.17 |
| Gao, 2017 | 722 | 0.71 | 20.50 | 16–25 | Northeastern | – | N | 0.32 |
| Xu & Sun, 2021 | 609 | 0.65 | – | 14–19 | Northeastern | MPATS | E & P & N | 0.07 & 0.28 & 0.23 |

Notes. MPATS = Mobile Phone Addiction Tendency Scale; Others = Others Scale; E = Extraversion; P = Psychoticism; N = Neuroticism.
